# Reduced user fees for antibiotics under age 5 in Hungary: Effect on antibiotic use and imbalances in the implementation

**DOI:** 10.1371/journal.pone.0219085

**Published:** 2019-06-28

**Authors:** Anikó Bíró

**Affiliations:** Health and Population “Momentum” Research Group at the Institute of Economics, Centre for Economic and Regional Studies of the Hungarian Academy of Sciences, Budapest, Hungary; Ottawa Hospital Research Institute, CANADA

## Abstract

**Objectives:**

In August 2016, new prescription guidelines were introduced in Hungary to reduce the co-payments for antibiotics among children aged 0-4. This study aims at analysing the implementation of this policy and its effect on the use of antibiotics.

**Methods:**

The analysis is based on administrative prescription records between January 2010—February 2018, covering the entire population of Hungary aged 0-7. Spatial autocorrelation indices are calculated and settlement level regression models are estimated to analyse the spatial variation in the application of the new guidelines. The effect of reduced co-payments on antibiotic use is estimated with a difference-in-differences type model: the treatment and control groups are children aged 0-4 and 5-7, respectively; the treatment and control periods are August 2016—February 2018 and January 2010—July 2016, respectively.

**Results:**

The new prescription guidelines are more widely applied in settlements with higher per capita income and lower unemployment rate. Adherence to the new guidelines is spatially clustered. A 10–15% decrease in the out-of-pocket costs of antibiotics is estimated to increase the consumption of antibiotics by about 5% (95% CI: 2.63%–7.55%).

**Conclusions:**

In the absence of clear enforcement mechanisms, the adoption of the new prescription guidelines is selective, contradicting the aims of the policy of making antibiotics affordable for the poor children. The results point to the possible role of physicians’ information networks in the application of prescription guidelines. The use of antibiotics among children aged 0-4 is responsive to the price subsidy of antibiotics.

## Introduction

In August 2016, a new prescription policy was introduced in Hungary. The social security subsidy rate of prescribed antibiotics doubled for children aged under 5 if the prescribing physician indicated specific (disease neutral) diagnosis codes on the prescription. However, prescriptions remained valid if the new guidelines were not followed, albeit the subsidy rate was lower in that case. Also, non-adherence to the new guidelines had no consequence for the physician.

This setting provides a unique opportunity to analyse the implementation of new prescription guidelines when a clear enforcement mechanism is missing. Thus, the first aim of this study is to analyse the adoption of the modified prescription guidelines by the physicians. We analyse if the adoption rate varies by settlement level socio-economic indicators, and if there is evidence for the importance of physician networks. This relates our study to the literature investigating the factors influencing the diffusion of a new drug ([1], [2], [3], among others) or a new technology, such as the electronic health records [4]. This literature points out the importance of physicians’ professional and social interactions in the uptake of medical innovations.

The second aim is to estimate how the reduced rate of user fees affects the use of antibiotics among children under the age of 5. There is evidence in the medical literature that the consumption of medication responds negatively to user fees ([5], [6], [7], [8], among others). Regarding the price elasticity of antibiotic use, there is much less evidence in the literature, and the existing results are mixed. Using the Rand Health Insurance Experiment, [9] document that cost-sharing has large impact on the use of antibiotics. On the other hand, using data from Italy, [10] find only weak albeit negative association between antibiotic consumption and co-payments.

The policy analysed in this paper makes it possible to estimate the response of antibiotic use to cost-sharing. We not only have data on antibiotic use pre and post the introduction of the new cost-sharing arrangements, but due to the nature of the new guidelines, there is a strict age cut-off in the applicability of the decreased cost-sharing. This quasi experimental setting naturally provides a treatment and a control group, and due to the allocation being dependent on age, we do not have to worry about endogenous selection into treatment.

## Policy background

The Hungarian health insurance system is employment based. Children up to age 18 are automatically insured. When purchasing medications, user fees are generally required. The user fees for medications depend on the magnitude of subsidies from the National Health Insurance Fund Administration, which greatly varies across substances. In case of antibiotics prescribed for children aged 0-7, and before the analysed policy, the basic subsidisation rate of 25% applied.

From August 2016, the subsidy of antibiotic purchases is raised to 50% for children aged under 5, if the ICD (International Statistical Classification of Diseases) codes Y40 (systemic antibiotics) or Y41 (other systemic anti-infectives and antiparasitics) are written on the prescription. The stated aim of the policy was to facilitate the affordability of antibiotics for the children of poor families. In principle, the prescribing physicians should apply the modified prescribing guidelines (i.e. the indication of the Y40, Y41 codes on the prescription) for all patients aged under 5, irrespective of the patient’s socio-economic status or other characteristics. However, the prescription is still valid and can be used if another ICD code is indicated on it. This implies that after the introduction of the policy, the prescribing physician has influence on the out-of-pocket costs of antibiotics in the affected age group.

## Materials and methods

### Data

We use administrative data on the purchases of antibiotics between January 2010—February 2018. The anonymised data cover the entire population of Hungary. The data were provided by the National Healthcare Services Centre (NHSC) of Hungary through an agreement between the NHSC and the Institute of Economics, Centre for Economic and Regional Studies of the Hungarian Academy of Sciences. The data cover pharmaceuticals in the ATC (Anatomical Therapeutic Chemical) group J01 (antibiotics with systemic use) that were purchased through pharmacies, hence relate only to the ambulatory setting and exclude hospital care. Only prescription based drugs are included in the data, which is not a limitation because antibiotics can be obtained in Hungary only based on prescription.

The data set includes the exact type of the antibiotic medication, the monthly date of the purchase, the amount purchased, the associated social security payment and out-of-pocket payment. Using these details, the days of therapy (DOT) can be attached to the data. The zip code of the patient’s address, the patient’s monthly date of birth and gender, and the ICD code of the diagnosis for which the prescription was given are also included.

Based on these records, a settlement and age specific monthly panel data of antibiotic purchases is constructed. When estimating the effect of the prescription guidelines on antibiotic use, we construct a country level, monthly date of birth specific monthly panel data. The pre-index (or baseline) period is January 2010—July 2016, while the post-index period is August 2016—February 2018. In accordance with the target age of the analysed policy, we restrict the sample to ages 0-7, with ages 5-7 serving as control group. The average population size in our sample of antibiotic users is 393,519 in the treatment group (age 0-4) and 280,144 in the control group (age 5-7).

Settlement level annual indicators of income, unemployment and the type of settlement originate from the T-STAR municipal statistical system of the Central Statistical Office of Hungary.

### Estimation methods

#### Analysis of prescription patterns

To capture to what extent physicians apply the new prescription guidelines in settlement *s* at monthly time *t* among patients aged *a*, we construct the following indicator:

$$\begin{array}{c} R_{a,s,t}=\frac{\sum (\text{antibiotic}\;\text{DOT,}\;\text{ICD = Y40}\;\text{or}\;\text{Y41)}}{\sum (\text{antibiotic}\;\text{DOT})}|age=a,settlement=s,time=t. \end{array}$$ (1)

We analyse how this ratio varies by age after the introduction of the new prescription guidelines (i.e. since August 2016), and by substance groups. When doing so, we add up the antibiotic DOTs over the entire period of August 2016—February 2018, both in the numerator and denominator of Eq (1). Then, we analyse the time pattern of the *R* ratio and the fraction of other ICD codes indicated on antibiotic prescriptions within the affected population, i.e. patients aged 0-4.

We also calculate and depict the micro-regional average of the *R* ratio throughout August—December 2016 and January—December 2017, restricting the analysis again only to the affected population. We also analyse on the settlement level, if similar values of the application rate of the new prescription guidelines are spatially clustered. If high application rate settlements are clustered together then that suggests the importance of professional and/or social networks in the flow of information regarding the new prescription guidelines. Using the *spatgsa* Stata command of [11], we calculate two global indices of spatial autocorrelation:

$$\text{Moran}’\text{s}\;\text{I}=\frac{\sum_{i=1}^{N}\sum_{j=1}^{N}w_{ij}Z_{i}Z_{j}}{S_{0}v},$$ (2)

$$\text{Geary}’\text{s}\;\text{C}=\left(N-1\right)\frac{\sum_{i=1}^{N}\sum_{j=1}^{N}w_{ij}{\left(Z_{i}-Z_{j}\right)}^{2}}{2NS_{0}v},$$ (3)

where *N* is the number of settlements, *w* are the elements of the spatial weights matrix (using 5 nearest neighbours), *Z* are the standardised values of the settlement specific indices of the application rate of the new guidelines (*R*), *S*_0_ is the total sum of the *w*s, and *v* is the sample variance of *Z*. Under no spatial autocorrelation, Moran’s I index takes the value 0. Values above 0 indicate positive spatial autocorrelation. In contrast, under no spatial autocorrelation, Geary’s C index equals 1, and values below 1 indicate positive spatial autocorrelation. More details on the indices are provided by [11].

A linear settlement level regression is estimated to investigate what observable factors can explain the spatial variation in the application of the new guidelines. Here the outcome variable is:

$$\begin{array}{c} \begin{array}{cc} R_{s}= & \frac{\sum (\text{antibiotic}\;\text{DOT,}\;\text{ICD = Y40}\;\text{or}\;\text{Y41})}{\sum (\text{antibiotic}\;\text{DOT})}|\\ & age=0-4,settlement=s,time=Aug2016-Feb2018, \end{array} \end{array}$$ (4)

and the estimated equation is:

$$\begin{array}{c} R_{s}=\beta_{0}+\beta_{1}I_{s}+\beta_{2}U_{s}+\beta_{3}type_{s}+\epsilon_{s}, \end{array}$$ (5)

where *I* is the settlement level average of the per capita taxable income over August 2016—February 2018, *U* is the average unemployment rate over the same period, and *type* is the type of the settlement (capital city / town / village).

#### Difference-in-differences analysis of the effect of the increased subsidy on antibiotic use

We collapse the data to a monthly country level panel of monthly cohorts. This construction of the data set allows us to control for cohort specific fixed effects when estimating the effect of the modified prescription guidelines on antibiotic use.

The logarithm of the monthly cohort *c* specific consumption at calendar month *t* is used as outcome variable (*logY*_*ct*_). Due to the lack of monthly age specific population statistics, we cannot reliably calculate monthly per capita consumption. We estimate the following difference-in-differences model:

$$\begin{array}{c} logY_{ct}=\alpha_{0}+\alpha_{1}D_{ct}+\alpha_{2}age_{ct}^{2}+\alpha_{3}age_{ct}^{3}+\tau_{t}+\chi_{c}+\varepsilon_{ct}. \end{array}$$ (6)

The sample is restricted to individuals aged 0-7, with children aged 5-7 belonging to the control group (unaffected by the new prescription guidelines). *D* is the binary treatment indicator, which equals one during the post-index period, that is between August 2016—February 2018 for cohorts aged 0-4. Thus, in this model, *α*_1_ is the treatment effect. We also include in the model a cubic polynomial of age, a full set of monthly date dummies (*τ*) and cohort dummies (*χ*).

We conduct negative control analyses with respect to time and age as follows. First, we assume that the same policy was introduced in August 2015. Here, to avoid the mixture of the consequences of the negative control with the real treatment, we cut the time horizon of the sample at July 2016. Second, we assume that treatment took place at the time of its real introduction (August 2016), but the treatment group consists of individuals aged at least 5 but less than 6, using those aged 6-7 as the control group.

We also analyse the heterogeneity of the estimated effect with respect to the settlement level unemployment rate (below/above the median), taxable income per capita (below/above the median) and settlement type. When doing so, we use a settlement and monthly cohort specific monthly panel data, and estimate Eq (6) extended with interaction terms between the treatment indicator *D* and the indicators of unemployment, income and settlement type. We weight these models with settlement level population size, the calculation of median unemployment rate and income is also based on weighted data.

## Results

### Application of the new prescription guidelines

**Fig 1:**
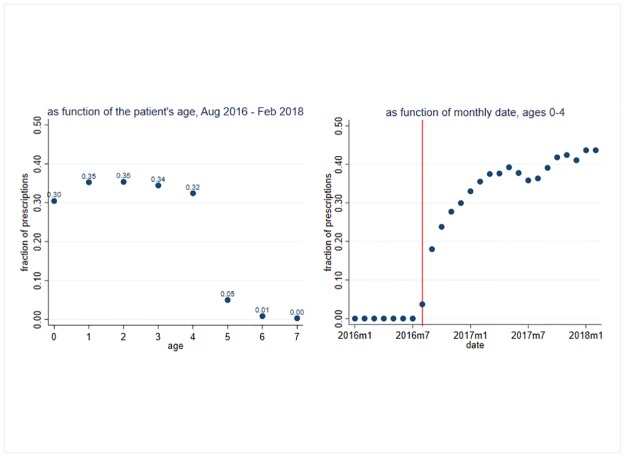
Application of the new guidelines by patient age and calendar time. Fraction of antibiotic prescriptions (measured in DOT) prescribed in line with the new guidelines.

Fig 1 shows that before the introduction of the policy, and in case of patients aged 5 and above, the Y40–Y41 ICD codes were not used for antibiotic prescriptions. The application rate of the new prescription guidelines increased gradually. By the end of the observation period, around 42% of the antibiotic prescriptions in the target age group were prescribed in line with the new guidelines.

**Fig 2:**
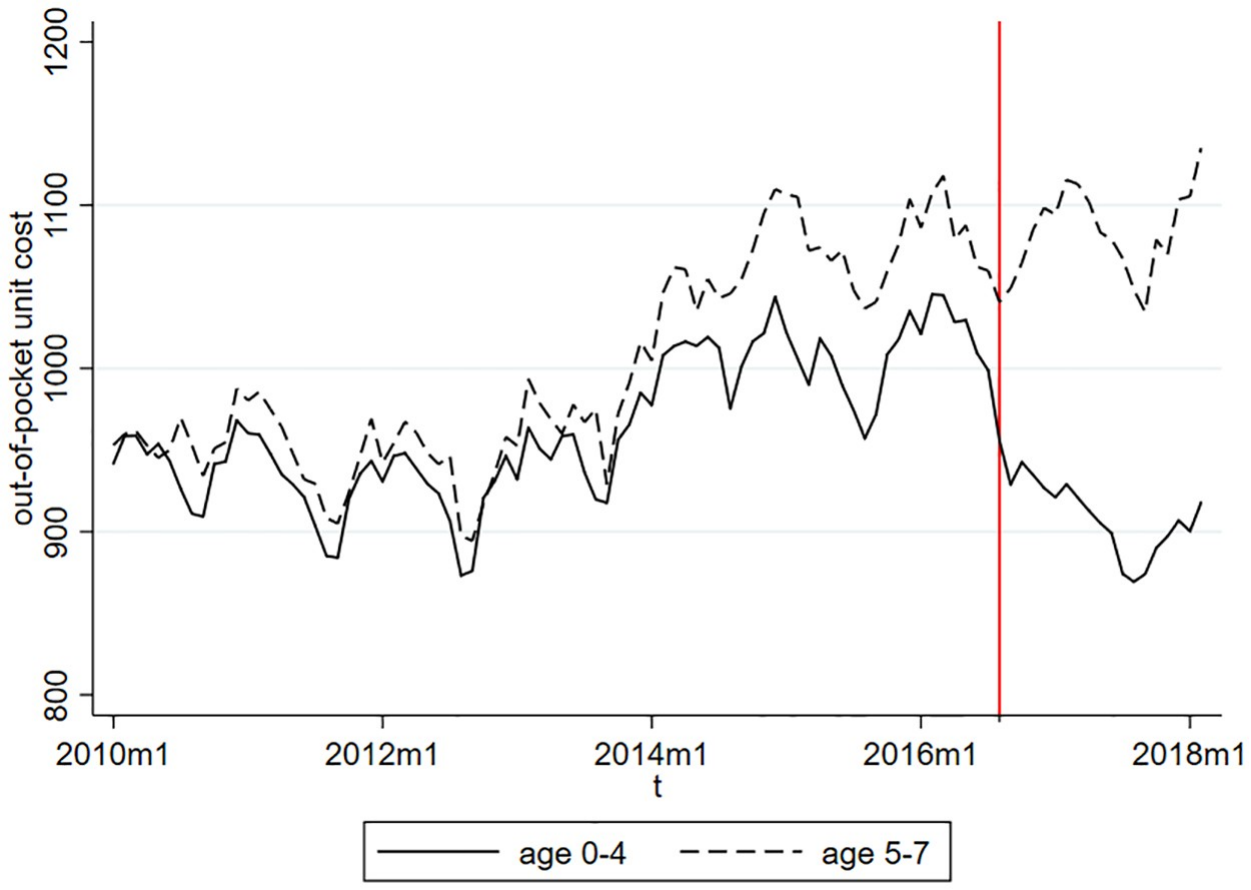
Average out-of-pocket unit cost (in HUF) of antibiotics by age groups.

Fig 2 shows that the unit out-of-pocket cost of antibiotics increased remarkably in 2014. Since then, the average out-of-pocket price per box of antibiotics is around 1, 050–1, 100 HUF (roughly 3.7 EUR) for children aged 5-7 (i.e. children not affected by the subsidisation policy change). The antibiotics prescribed to younger children are about 5% cheaper. After the introduction of the new prescription guidelines, the unit out-of-pocket cost among children aged 0-4 fell on average by around 10–15%, while the out-of-pocket cost among children aged 5-7 remained at the pre treatment level.

Table 1 shows for the most prevalent types of antibiotics the ratio prescribed in line with the new prescription guidelines, their average out-of-pocket (OOP) unit price and their fraction within the total antibiotic prescriptions. There is some heterogeneity across substances, the new guidelines seem to be applied to a higher extent in case of more expensive antibiotics.

**Table 1:** Prescription patterns by ATC groups, ages 0-4, August 2016-February 2018.

	fraction prescribed with ICD codes Y40, Y41	fraction within total antibiotic DOT	average unit OOP price (HUF)
amoxicillin	0.373	0.086	344.9
amoxicillin and beta-lactamase inhibitor	0.362	0.432	740.4
azithromycin	0.401	0.102	1341.8
cefaclor	0.256	0.039	822.8
cefixime	0.425	0.071	1159.2
cefprozil	0.450	0.075	1632.5
cefuroxime	0.279	0.055	888.2

**Fig 3:**
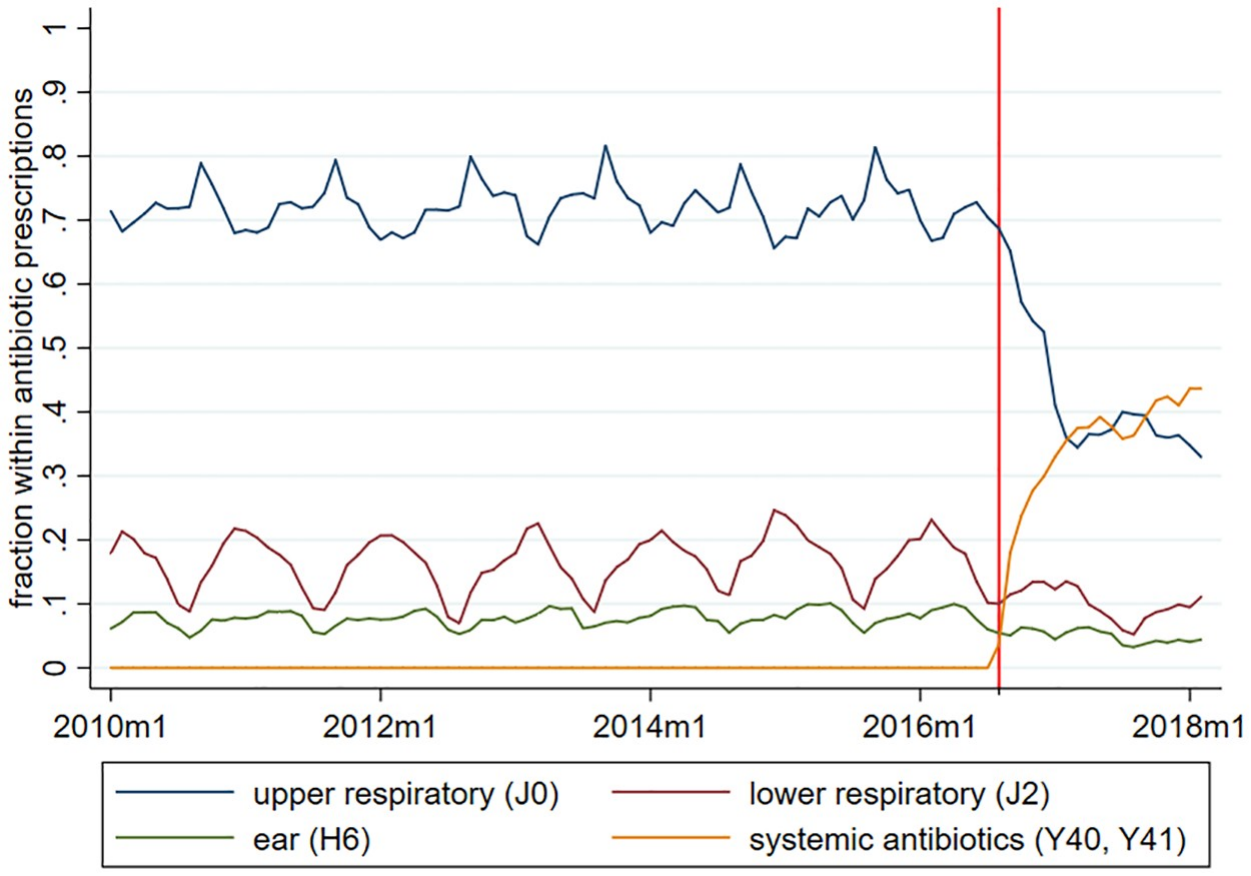
Distribution of antibiotic prescriptions by ICD groups, ages 0-4. The prescriptons are measured in total DOT.

Next, Fig 3 depicts the time patterns of the most prevalent ICD codes indicated on antibiotic prescriptions, ages 0-4. Before the introduction of the new prescription guidelines, about 70–80% of antibiotic prescriptions were prescribed for upper respiratory diseases. After August 2016, the indication of upper respiratory diseases on antibiotic prescriptions fell to around 35–40%. Thus, essentially, the newly applied diagnosis codes Y40–Y41 replaced the ICD codes of upper respiratory diseases. The prevalence of ICD codes of ear and lower respiratory diseases also decreased, but due to the lower baseline levels, these decreases were less substantial in absolute value.

Table 2 indicates that the application rate of the new guidelines is higher in more affluent settlements, as measured by settlement level average taxable income and unemployment rate. It is also higher in towns than villages.

**Table 2:** Settlement level linear regressions of the rate of application of the new prescription guidelines, August 2016-February 2018.

	application rate of new guidelines	
	ages 0-4	ages 3-4
taxable annual income per capita, million HUF	0.102*** [0.0520; 0.1520]	0.0762*** [0.0243; 0.1281]
unemployment rate	-0.482*** [-0.8172; -0.1468]	-0.601*** [-0.9381; -0.2639]
settlement type (baseline: capital city) town	0.0928*** [0.0671; 0.1185]	0.0916*** [0.0659; 0.1173]
village	-0.0118 [-0.0291; 0.0055]	-0.00518 [-0.0234; 0.0131]
constant	0.265*** [0.2033; 0.3267]	0.286*** [0.2221; 0.3499]
observations	2,845	2,759

95% confidence intervals in brackets,

*** p<0.01,

** p<0.05,

* p<0.1

**Fig 4:**
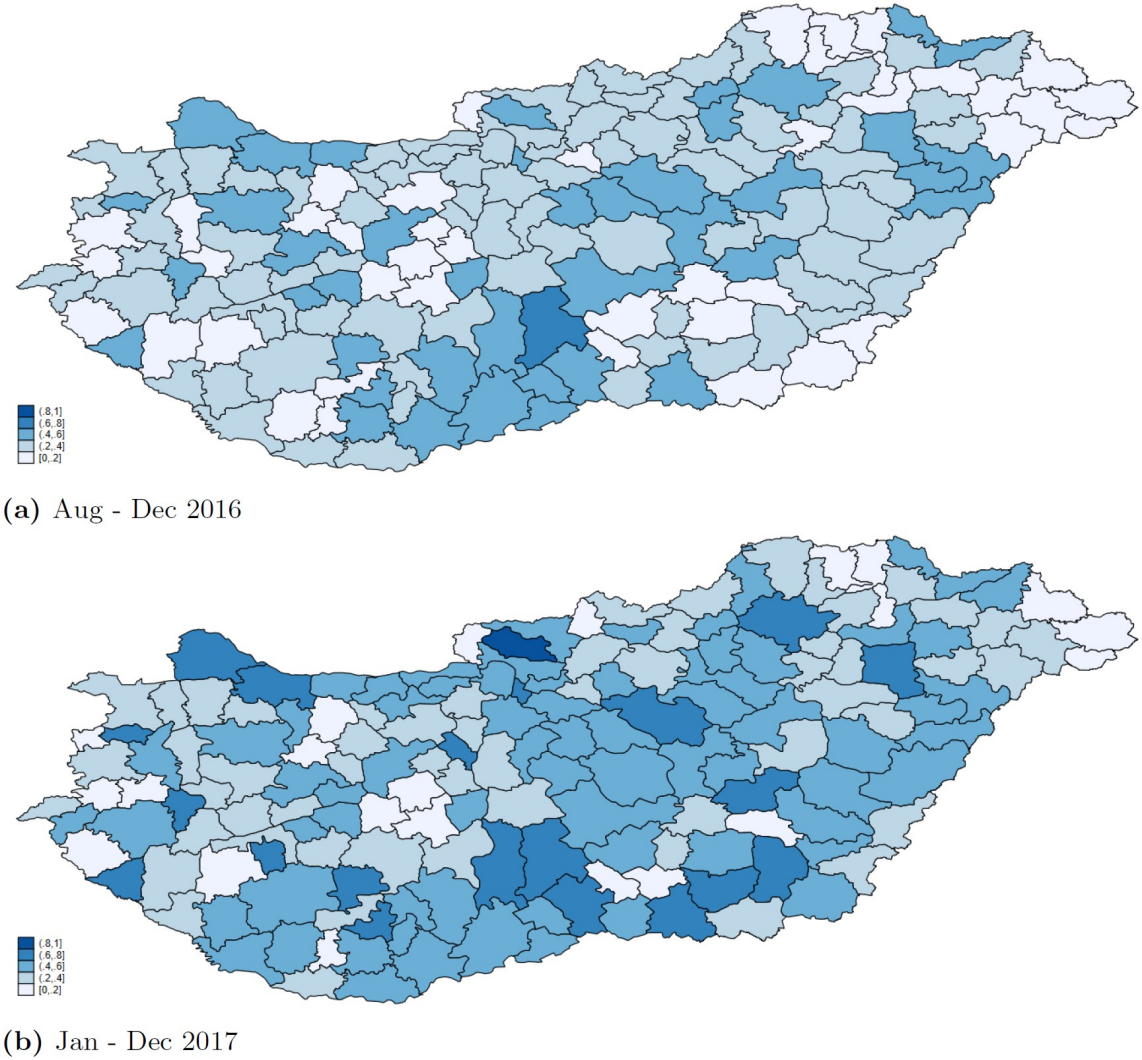
Regional variation in prescription patterns. Fraction of antibiotic prescriptions (measured in DOT) prescribed in line with the new guidelines by micro regions, patients aged 0-4.

Fig 4 shows that both in 2016 and 2017, there were blocks of micro-regions where the prevalence of the application of the new guidelines was similarly high (low) in neighbouring micro-regions. The spatial clustering is confirmed by the calculation of global indexes of spatial autocorrelation at the settlement level: Moran’s I is positive, and Geary’s C is less than 1. Looking at year 2017, the Moran’s I statistic of spatial autocorrelation is 0.247 (p-value 0.000), and the Geary’s C statistic of spatial autocorrelation is 0.759 (p-value 0.000).

### Antibiotic use

**Fig 5:**
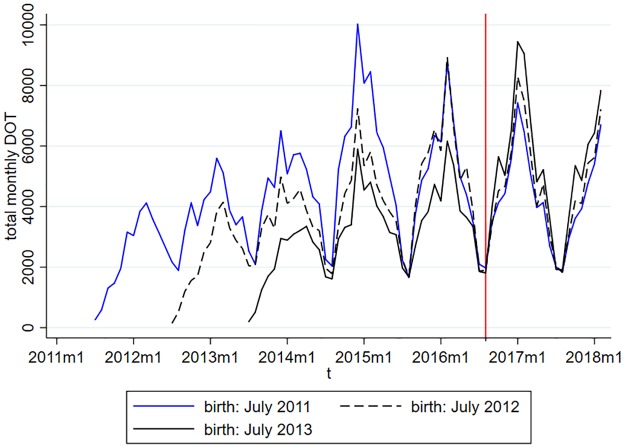
Country level time patterns of total antibiotic DOT for selected monthly cohorts.

Fig 5 shows the time pattern of antibiotic consumption for a cohort not affected by the new prescription guidelines (born in July 2011) and two cohorts affected by the guidelines (born in July 2012 and July 2013). Antibiotic consumption is strongly seasonal, the Winter peak of the consumption is up to four times higher than the consumption in July and August. Due to the effect of ageing, the effect of the price subsidies introduced in August 2016 cannot be easily figured out from Fig 5.

Table 3 shows average values of antibiotic DOT before and after the introduction of the new prescription guidelines, by ages below and above age 5. These calculations indicate that the policy had a positive effect on antibiotic use; the difference-in-differences is positive, with an increase in use equal to 4% of the antibiotic use in the treatment age group (ages 0-4) before the implementation of the policy. However, these calculations do not net out the calendar time and cohort effects. In the following, we estimate precisely the consumption effect of the new prescription guidelines. Table 3 also indicates that antibiotic use decreased in the analysed period (January 2010—February 2018) both in the age groups 0-4 and 5-7, however, the decrease in usage was stronger in the age group unaffected by the subsidisation policy (ages 5-7).

**Table 3:** Antibiotic use by age group and time periods. Average values of monthly cohort specific antibiotic DOT per calendar month.

	Jan 2010-Jul 2016 (“before”)	Aug 2016-Feb 2018 (“after”)	“after”-“before”
age 0-4	4073.6 [4014.1; 4133.1]	3455.0 [3336.5; 3573.4]	-618.7 [-757.5; -479.8]
age 5-7	4508.2 [4432.5; 4583.9]	3736.1 [3601.3; 3870.9]	-772.1 [-940.1; -604.1]
			difference-in-differences 153.4 [-82.7; 389.5]

95% confidence intervals in brackets

**Fig 6:**
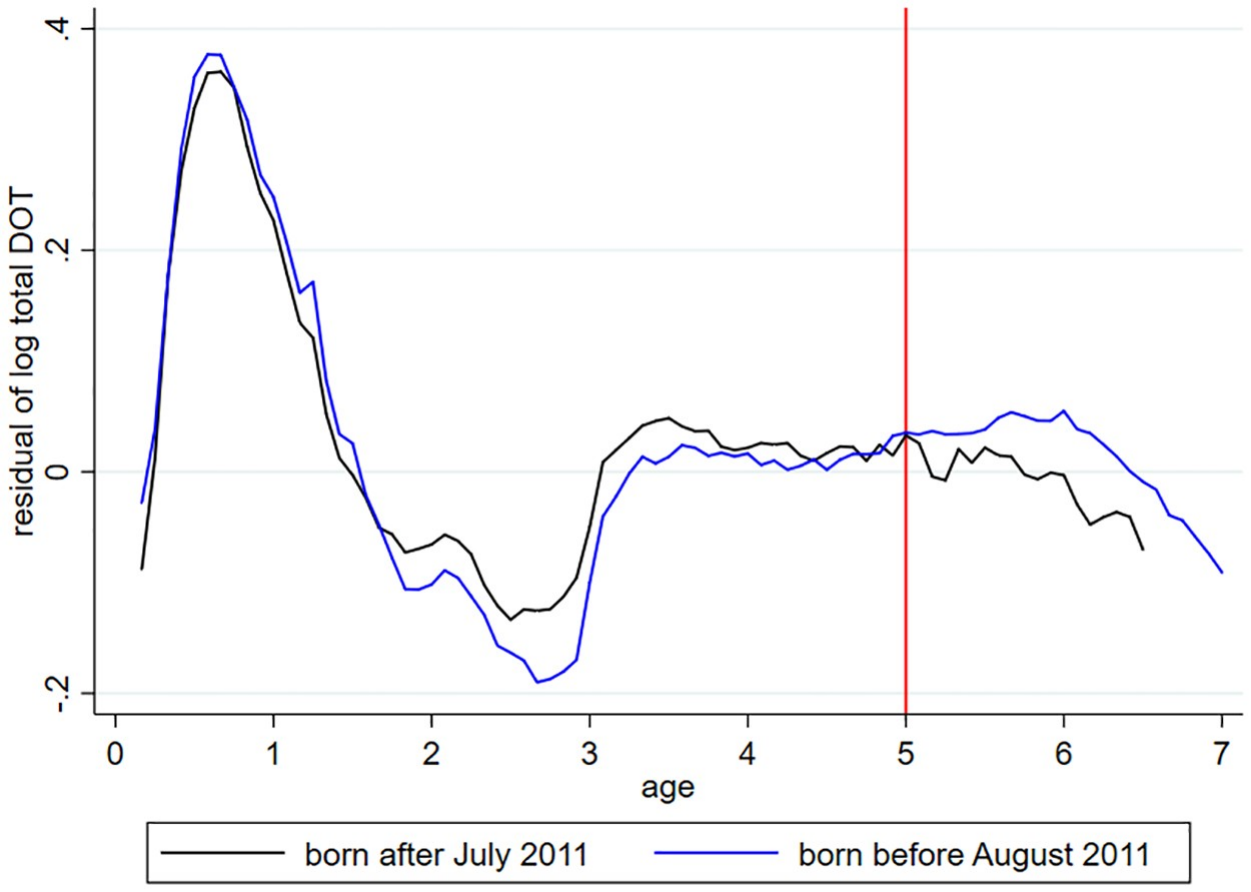
Residuals of the logarithm of antibiotic DOT as function of age, by cohort groups. The residuals are calculated from Eq (6).

Fig 6 plots the average residuals from Eq (6) by age. Logarithmic antibiotic consumption unexplained by cohort, calendar month and cubic age effects is on average higher among children who were affected by the new prescription guidelines (i.e. born after July 2011), but only up to age 5, which is the age above which the new guidelines are not applicable.

Table 4 shows the estimation results of Eq (6). These results suggest that the 10–15% decline in the out-of-pocket cost of antibiotics resulted in about 5.1% increase in the use of antibiotics among children aged 0 to 4, over a 19 months long follow-up period (95% CI: 2.63%—7.55%). The estimated effects under the negative controls are smaller and negative. Assuming that the policy was introduced a year earlier gives an estimated effect of -1.2% (95% CI: -6.2%–+3.8%). Assuming that the policy affected children aged at least 5 but less than 6 gives an estimated effect of -0.4% (95% CI: -2.3%–+1.4%). Thus, the negative control estimates support that the main estimate indeed shows the effect of the increased subsidisation of antibiotic use among children age 0-4.

**Table 4:** Estimated effect of the increased price subsidy on antibiotic use. Baseline results and negative control analyses.

	log DOT	negative control: timing, log DOT	negative control: age, log DOT
treatment effect	0.0509*** [0.0263; 0.0755]	-0.0121 [-0.0620; 0.0378]	-0.0044 [-0.0225; 0.0137]
age squared and cubic	yes	yes	yes
monthly date dummies	yes	yes	yes
monthly cohort fixed effects	yes	yes	yes
number of month × cohort cells	7.834	6,637	2,450

95% confidence intervals in brackets,

*** p<0.01,

** p<0.05,

* p<0.1

The outcome measure is the logarithm of total antibiotic DOT per monthly cohort and calendar month.

The results of Table 5 show that the positive effect of the new prescription guidelines is present only in settlements with lower unemployment rate and is also stronger in settlements with higher per capita taxable income. Also, the estimated effect is the highest in towns. These results are in line with the evidence documented in Table 2 that the new prescription guidelines were more widely applied in the more affluent settlements and in towns.

**Table 5:** Heterogeneity in the effect of the increased price subsidy on antibiotic use by settlement level indicators.

	log DOT, heterogeneity by		
	unemployment	income	settlement type
treatment × below median unemployment rate	0.0693*** [0.0525; 0.0861]		
treatment × at or above median unemployment rate	0.0039 [-0.0098; 0.0176]		
treatment × below median income per capita		0.0133* [-0.0004; 0.0270]	
treatment × at or above median income per capita		0.0535*** [0.0380; 0.0690]	
treatment × capital city			0.0226 [-0.0072; 0.0525]
treatment × other town			0.0714*** [0.0582; 0.0846]
treatment × village			0.0578*** [0.0474; 0.0682]
age squared and cubic	yes	yes	yes
monthly date dummies	yes	yes	yes
monthly cohort × settlement fixed effects	yes	yes	yes
number of month × settlement × cohort cells	3,788,569	3,788,569	3,788,569

95% confidence intervals in brackets,

*** p<0.01,

** p<0.05,

* p<0.1,

regressions weighted by settlement population size. The outcome measure is the logarithm of total antibiotic DOT per settlement, monthly cohort and calendar month.

## Discussion

We analysed the adoption of new prescription guidelines aimed at reducing the co-payments for antibiotics among children aged 0-4 in Hungary. Around 42% of the prescriptions are issued in line with the new guidelines 19 months after the introduction of the policy. This result clearly shows that if there are no clear enforcement mechanisms, the adoption of prescription or other, similar guidelines is selective.

The results of the spatial analysis point to the possible role of physicians’ information networks in the application of the new guidelines. We found evidence for spatial clustering in the application of the new prescription guidelines. This could not have been policy driven, as the guidelines are supposed to be applied for all antibiotic prescriptions of patients aged under 5. Since the prescribing physicians do not have monetary incentives against the application of the new prescription guidelines, and also due to the specific nature of the guidelines (indication of special ICD codes on the prescription), the spatial clustering in the adherence to the new guidelines is likely due to the spread of information among physicians.

Settlement level analysis reveals that the new prescription guidelines are more widely applied in the better-off settlements, possibly because physicians working in these areas are more knowledgeable about the latest prescription guidelines. A 1% point lower unemployment rate is associated with around 0.5% point higher application rate of the new prescription guidelines. A 10% higher per capita taxable income at the mean (corresponding to a higher income by around 70,000 HUF) is associated with around 0.7% point higher application rate of the new prescription guidelines. The application rate is 9% points lower in villages than in rural towns. Overall, apart from the differences by settlement type, the differences in the adoption of the new guidelines by settlement level economic indicators are moderate. Still, these results suggest that the equity aims of the policy are violated by selective adoption.

The estimated effect of cost-sharing on antibiotic consumption is in line with earlier evidence in the literature regarding the out-of-pocket price elasticity of the consumption of prescription drugs. For example, according to the meta-analysis of [7], for each 10% increase in cost sharing, prescription drug spending decreases by 2% to 6%. In line with these results, we find about 5% increase in antibiotic consumption among children aged 0-4 due to a 10–15% decline in the out-of-pocket cost of antibiotics.

The current study is subject to some limitations. Due to the nature of the administrative data used, we cannot analyse among others if and how the prescribing physicians’ or the patients’ characteristics affect the adoption of the new prescription guidelines. The external validity of the results regarding the adoption patterns of the new guidelines is restricted due to the specific nature of the policy.

The regression models of antibiotic use were estimated on data aggregated to monthly cohort, monthly calendar date and settlement cells. Since the application of the treatment varies by birth of date and calendar time, this approach does not affect the estimated effect of the price subsidy. However, due to data limitations, we cannot estimate the effect of individual level income or labour force status on the subsidy rate and use of antibiotics. Instead, we use settlement level average income and unemployment rate indicators. This approach does not bias the results if the application of the new prescription guidelines does not vary by the socio-economic status of patients within a settlement.

Overall, we provided evidence that not only the implementation of new technologies are selective and depend on physicians’ networks (as known from the literature), but also the adoption of guidelines such as the one analysed in this study related to the prescription of antibiotics. Less developed regions are likely not to enjoy the benefits of the new guidelines (or innovations) if physicians are not perfectly informed about policy changes or innovations and if there are no or imperfect enforcement mechanisms. We also conclude that the use of antibiotics among children aged 0-4 is responsive to the price subsidy of antibiotics. This finding is particularly important if the reduction of antibiotic overuse is a policy aim.
